# Development of a theory-based HPV vaccine promotion comic book for East African adolescents in the US

**DOI:** 10.1186/s12889-021-11005-2

**Published:** 2021-06-14

**Authors:** Isabelle Celentano, Rachel L. Winer, Sou Hyun Jang, Anisa Ibrahim, Farah Bille Mohamed, John Lin, Fanaye Amsalu, Ahmed A. Ali, Victoria M. Taylor, Linda K. Ko

**Affiliations:** 1 Department of Health Services, University of Washington School of Public Health, 1959 NE Pacific Street, Magnuson Health Sciences Bldg., Box 357660, Seattle, WA 98195 USA; 2 Department of Epidemiology, University of Washington, Box 359933, 325 9th Ave, Seattle, WA 98104 USA; 3 Department of Sociology, Sungkyunkwan University, Seoul, South Korea; 4 Department of Pediatrics, University of Washington, Harborview Medical Center, 325 9th Ave, Seattle, WA 98104 USA; 5 Somali Health Board, 625 Strander Blvd Building, Tukwila, WA 98188 USA; 6 Division of Public Health Sciences, Fred Hutchinson Cancer Research Center, 1100 Fairview Ave. N, M3-B232, Seattle, WA 98102 USA; 7 Department of Health Services, University of Washington and Division of Public Health Science, Fred Hutchinson Cancer Research Center, Hans Rosling Center for Public Health, 3980 15th Avenue NE, UW Mailbox 351621, Seattle, WA 98195 USA

**Keywords:** HPV vaccine, Behavioral theory, Comic book, East African adolescents, East African parents

## Abstract

**Background:**

Human Papillomavirus (HPV) vaccine uptake is low among East African adolescents in the US. Adolescents’ preferences influence HPV vaccine decisions, yet few interventions exist that address East African adolescents’ beliefs about HPV vaccines. We describe a multi-step process on how to create a theory-based comic book by integrating empirical findings, theory and focus group data from East African parents in the US.

**Methods:**

Our multi-methods process included conducting focus groups with Somali, Ethiopian, and Eritrean mothers (*n* = 30) to understand mothers and adolescents socio-cultural beliefs and information needs about the HPV vaccine, creating comic book messages integrating the focus group findings, and assessing the acceptability of the finalized comic book among Somali, Ethiopian, and Eritrean adolescents (*n* = 134).

**Results:**

We identified categories around socio-cultural beliefs (such ethnic representation and concerns about pork gelatin in vaccines), HPV vaccine information needs, and diffusion of information. We then mapped the categories to theoretical constructs and operationalized them into the comic book. Finally, we describe the overall acceptability of the comic book and specifics on comic book structure, appeal of characters, and message relevance.

**Conclusions:**

A rigorous multi-step process that integrates theory and focus group data can help create culturally appropriate health messages that can educate and appeal to the community.

**Supplementary Information:**

The online version contains supplementary material available at 10.1186/s12889-021-11005-2.

## Background

Given their appeal to educate and entertain at the same time, graphic novels or comic books have emerged as an effective health communication tool to promote behavior change [1]. Unlike text-only information, comic books depict text with visceral visual messages that enhance content understanding, facilitate connections between present knowledge and new information, and improve information recall [2, 3]. Given their wide appeal for children and adolescents, comic books have emerged as a preferred health communication tool for educating young audiences on public health issues in the US and other countries. Comic books have been reported to improve children’s knowledge and behaviors about back pain, burn safety, smoking cessation, nutrition and physical activity, and human papillomavirus (HPV) vaccine uptake [4–8].

In the US, routine HPV vaccination is recommended at age 11–12 years, with catch-up vaccination recommended through age 26 for anyone who has not been previously vaccinated [9]. Despite public health efforts, HPV vaccine uptake is suboptimal. A recent paper on HPV vaccine initiation and completion among 13–17 year old across four racial/ethnic groups reveals disparities for vaccine initiation (76% in Hispanics, 64% in non-Hispanic Whites, 73% in non-Hispanic Blacks, and 65% in non-Hispanic Asians) and completion (57% in Hispanics, 48% in non-Hispanic Whites, 53% in non-Hispanic Blacks, and 53% in non-Hispanic Asians) [10]. HPV vaccine uptake among East African communities is particularly low and highly understudied. A small mixed methods study examining adolescent vaccine (Pertussis/Tdap, Meningococcal/MCV4, and HPV) uptake among Somali, Ethiopian, Eritrean, and Hispanic mothers conducted in 2012 showed that none of 55 Somali mothers and only 8 out of 50 (16%) Ethiopian or Eritrean mothers reported vaccinating their children for HPV [11]. While more studies are needed to understand reasons for low HPV vaccine uptake in these communities, prior research in both Africa and in the US indicate that mistrust [12–14] and hesitancy for vaccines in general [15, 16], lack of awareness about HPV vaccine [11], and cultural beliefs about western medicine [17] contribute to suboptimal uptake.

Comic books may be a promising tool for communicating about HPV vaccines to East African adolescents. Parents, healthcare providers, and children all play a role in decisions to receive the HPV vaccine [11, 18–20]. While many interventions have focused on addressing parents’ concerns about the HPV vaccine [21, 22] and engaging providers to make strong vaccine recommendations [23], less has been done to address children’s concerns about the HPV vaccine. For East African communities, conversations between parents and children about HPV vaccination may be more sensitive as discussions can include information on sexual health, which is stigmatized in these communities [24, 25]. Comic books can be effective on addressing a sensitive topic with a light-hearted approach (compared to traditional print materials) as fictional characters embedded within a realistic setting such as families and institutions (school) can be portrayed in engaging in difficult conversations through humor, empathy, and other positive emotions [26].

While studies on comic books report using theory to develop the content of the story [4, 5], few, if any studies, have described the development process and how theory was integrated into the storyline. In this paper, we use the development of a HPV vaccine comic book for East African adolescents as a case study to illustrate such a process using the perspectives of parents and adolescents.

## Methods

We developed a comic book as part of a multilevel communication intervention for adolescents, mothers, and healthcare providers to promote HPV vaccination among East African adolescents [27]. The comic book was designed to appeal to and be appropriate for 14–17-year-old adolescents. Our multi-step process included 1) review of the literature on perceptions of HPV vaccine and uptake among East African mothers and their adolescent children, 2) focus groups with East African mothers to understand the socio-cultural beliefs and information needs about the HPV vaccine, and 3) integration of the information from the literature, the focus group data, and behavior change theories to develop theory-based messages for the comic book story. We then assessed the acceptability of the comic book as well as specifics on comic book structure, character appeal, and message relevance among East African adolescents. The University of Washington Institutional Review Board approved this study.

### Focus groups

We conducted focus groups with East African mothers in King County, Washington to inform the development of the larger multilevel communication intervention. The purpose of the focus groups was to understand mothers’ socio-cultural beliefs and information needs about the HPV vaccine and to gather input on a comic book mockup developed by the research team (see below for more description). Detailed recruitment, data collection tool, and data collection strategies for the focus groups were described previously [17]. Briefly, we convened three focus groups of 9–11 women each (*n* = 30) in Somali, Amharic, and Tigrinya. Women were eligible to participate if they were fluent in Somali, Amharic, or Tigrinya and had at least one 11–17 year-old child (inclusive of both the lower end of the target age for HPV vaccination and the target age range for the comic book intervention) [9]. We used purposive sampling to recruit mothers based on self-reported HPV vaccination status of their children, including up to 3 mothers with vaccinated children in each group; the inclusion of mothers with vaccinated and unvaccinated children helped us learn about both anticipated and experienced barriers to and facilitators of HPV vaccination.

#### Comic book mock-up

A half-page storyline for the comic book was created by the research team. The story considered previous literature on vaccine barriers and facilitators [4, 28] and was informed by two behavioral theories: Health Belief Model (HBM) [29] and Theory of Reasoned Action (TRA) [30]. Bilingual research team members translated the storyline from English into Somali, Amharic, and Tigrinya. Table 1 presents the storyline for each scene, which was superimposed onto photos of adolescents that resembled members of the target communities. Images were obtained from health promotion materials and inventories of publicly available stock photos. The comic book mock-up was reviewed for cultural relevance by East African community members and our bilingual/bicultural research team.

**Table 1:** Scenes and story line

Scene	Story line
Scene 1	The comic book follows an adolescent female main character (MC). Her health teacher invites a local doctor to talk about HPV and HPV vaccine to her class. The doctor tells the class that HPV is so common that almost everyone will be infected at some point – the scary part is they may never know, and it can cause cancer. The doctor urges students to get vaccinated for HPV.
Scene 2	After school ends, MC walks home with her friend who says that she got vaccinated for HPV a few weeks ago to prevent cancer. She mentions several other friends, both boys and girls, who got vaccinated. MC always disliked vaccination because she finds it painful. Her friend reminds her that it is a relatively easy pain compared to fighting cancer or even studying for their next math test.
Scene 3	When MC arrives home, she asks her mother to talk to her doctor about the HPV vaccine at their next visit because she wants to get vaccinated. After her mother and grandmother learn about the benefit of the vaccine to prevent cancer, she agrees that it is a very good idea to get vaccinated and is very proud of her daughter’s decision.

#### Focus group data collection

The focus group moderator guide included questions on socio-cultural beliefs around HPV vaccines and specific questions about the comic book mock-up on storyline content, featured characters, overall graphic design, and cultural relevance of the comic book mock-up. Examples of questions about the mock-up included ‘How clear was the information presented in the material?’, ‘What are your thoughts about the characters, the story, and the setting?’, ‘Should other family members be included as characters?’, ‘If so, who should be included?’, and ‘How do we make this material more culturally appropriate to your community and to the adolescents?’ Focus group sessions were conducted in Somali, Amharic, or Tigrinya, audio recorded and translated from Somali, Amharic, or Tigrinya into English.

#### Focus group data analysis

Data analysis involved three steps. First, researchers met after each focus group, developed notes on key themes, and provided feedback to the moderator for the next focus group to clarify emerging themes. Second, two researchers independently reviewed each transcript and used inductive, constant comparison approach to identify concepts. Using an iterative process, the researchers met biweekly to refine the codebook, adding, removing, and revising codes as needed to address inter-rater agreement and to compare new codes with existing codes. Third, we reached consensus around themes identified throughout the coding and analysis process. The data were organized using ATLAS.ti, version 7.

### Message mapping

Message mapping was used to operationalize the inputs received on the HPV and HPV vaccine related messages presented in the mock-up during the focus group. Message mapping is a method that we developed to integrate literature review, our own focus group findings, and theory to create theory-based messages. The development of the message mapping method was informed by intervention mapping, a planning method that provides a system for integration of theory, empirical findings from the literature, and information collected from the target population [31] to develop culturally-appropriate and theoretically-sound interventions.

We reviewed the literature on strategies used to increase vaccine uptake for parents, healthcare providers, and children in general and specifically for East African communities. A summary of this review is beyond the scope of this paper. However, within the health behavior literature we found descriptions on theory-informed messages around HPV vaccine uptake developed with input from parents [4, 32]. Commonly used theories were HBM and TRA. Informed by this review, we created messages that align with six constructs of HBM (perceived susceptibility, severity, benefits, barriers, self-efficacy, and cues to action) and two constructs from TRA (behavioral intention and subjective norm). These messages were integrated into the mock-up presented during the focus group to assess message relevance and cultural appropriateness.

After the focus groups, messages were revised to include the input from the mothers. For example, the mock-up did not include information on pork gelatin (identified as a barrier for vaccine uptake during the Somali focus group), thus, this was added into the final comic book. Some messages, framed more generally in the mock-up, were revised to be specific; for example, the behavioral intention message “I plan to get the HPV vaccine now,” was revised to “I know grandma. That’s why I think I need to get it now.” Another change included replacing the character who delivered the message. For example, the subjective norm message was originally delivered by the main character’s mother as “You even told me that many of your friends have had the HPV vaccine shots,” but after the focus group, the message was revised to be delivered by the main character’s peers as “Oh yeah! I totally got vaccinated in the beginning of the school year. I know Ayan and Fatima also got vaccinated.” The theoretical constructs, the message objectives and the final comic book messages are described in Table 2. The final comic book content was reviewed by the community partners before moving to production.

**Table 2:** Theory-based messages included in the comic book

Theoretical constructs	Message objective	Operationalized in the comic book
Perceived susceptibility	Increase perceived susceptibility	“HPV is so common that almost everyone will be infected at some point.” “Most people infected will never know.”
Perceived severity	Specify consequences of the risk and condition	“… cause disease and trouble like cancer.”
Perceived benefits	Define action to take; clarify the expected positive effects	“The vaccine will protect you from cancer in the future.” “so we will not get sick in the future from something we could have prevented.” “It’s an easy step that you can take now to prevent cancer in the future.”
Perceived barriers	Identify and reduce barriers through reassurance, incentives, assistance	“I’ve always hated shots because they hurt.” “No grandma, the doctor said that it does not have pork gelatin.”
Cues to action	Provide how-to information; promote awareness; give reminders	“Talk to your parents, nurses, and doctors about the HPV vaccine.”
Self-efficacy	Provide training and guidance in performing action	“… I am sure I can ask my parents to talk to my doctor about the HPV vaccine.” “… I am sure I can get the HPV vaccine.”

### Acceptability of the comic book

#### Recruitment

From October 2017 to September 2018, mothers and their adolescent children were recruited to participate in ethnolinguistic-centric educational dinners on HPV vaccination. Dinners included both an interactive educational forum intervention for mothers (to promote HPV vaccination in their 11–17-year-old children) and the comic book intervention for 14–17-year-old adolescent children. Dinners were held in Somali or Amharic only; no dinners were held in Tigrinyan due to insufficient staffing to support recruitment and data collection, and therefore we did not evaluate acceptability of the Eritrean comic book version. Mothers were eligible to participate if they spoke Somali or Amharic fluently or very well, had ≥1 child aged 11–17 years, and reported that all of their children were either unvaccinated against HPV or that they did not know if their children had been vaccinated. Mothers were invited to bring ≥1 child aged 14–17 years to participate in the comic book intervention if the child was able to speak and read English fluently or very well. Bilingual staff members recruited mothers face-to-face at community events. Our community partners also provided contact information for potential participants for telephone recruitment. The staff member explained the study in the woman’s native language and collected children’s age, gender, and HPV vaccination status. Additional demographic and immigration information were collected for participating women. Mothers provided written informed consent in their native language on behalf of themselves and their participating children. Children provided verbal assent. At the events, mothers and adolescents ate dinner together before participating in separate educational activities on HPV vaccines. Mothers participated in an interactive native language presentation led by a bilingual co-ethnic health care provider, while adolescents read the comic book. Adolescents and their mothers each received $25 for participating.

#### Data collection

Each participating child was given a copy of the comic book and asked to respond to four open-ended questions (Additional file 1) after reading the comic book: 1) adolescents’ thoughts about the comic book in general, 2) what adolescents liked and did not like about the comic book, 3) their thoughts on whether other adolescents would like the comic book and why, and 4) what messages in the comic book were important to them.

#### Data analysis

The research team conducted content analysis, and excerpts were counted of the acceptability data. For the content analysis, two research team members independently reviewed each transcript to identify major categories and codes and created a tentative coding scheme. The researchers refined the coding scheme throughout data analysis. Discrepancies were resolved via reconciliation. Once the content analysis was completed, the research team counted the number of excerpts identified under the major categories and codes to calculate the frequency and the proportion of the excerpts.

## Results

### Demographics of the focus group participants

Participants’ mean (standard deviation) age was 41.0 (5.6) years, and they had 9.5 (4.5) years of formal education. All mothers were born outside of the US; most were married (80%); some (20%) had at least one child between the ages of 11–17 who was vaccinated for HPV. Detailed demographic data have been previously reported [17].

### Comments about the comic book mock-up

We identified four major themes on how to improve the comic book based on the mock-up. These include mothers’ preferences on the comic book characters, information needs of mothers and adolescents, appeal of the messages to the mothers and the adolescents, how mothers perceived diffusion of information in their community, and the impact of social influence on adolescents’ vaccine decisions (Table 3).

**Table 3:** Development of the comic book structure and content

Themes	Participant comment	Operationalized in the comic book
*Characters*	• Characters should look like the target population, and should be in appropriate, modest dress	• Characters wearing hijab for Somali comic book and non-hijab wearing characters for Eritrean and Ethiopian comic books; names of the characters are from the community for all three comic books
	• Include girls and boys as characters	• Two boys in Scene 2
	• Include a grandmother	• A grandmother in Scene 3
	• Mother represents the whole family	• Mother and grandmother in Scene 3
*HPV information*	• Include information about HPV knowledge (transmission, symptoms, and consequences) • Differentiate HPV from HIV	• Spread through sexual contact
		• Most won’t know they are infected because there are no symptoms • The types of cancer caused by HPV
		• A student mistakes HPV for HIV when asked in class, and doctor emphasizes the ‘P’ in HPV when written on the board
*HPV vaccine information*	• Include information about cancer prevention	• Grandma approves the vaccine and states that there were no such vaccines to prevent cancer when she was young
	• Include information about early vaccination	• A doctor tells the students to get the vaccine now to prevent cancer later by comparing the HPV vaccine to putting on a seatbelt before driving
	• Include information about side effects	• A doctor mentions common side effects of the vaccine, and that the vaccine has been shown to be safe from serious or long-term side effects
*Social influence*	• Friends play an influential role to each other	• Main character discusses the vaccine with her friends who have already been vaccinated and they encourage each other to discuss the vaccine with their parents.
	• Adolescents get information from multiple sources, not just from parents	• HPV and HPV vaccine information is disseminated by a member of the health department (Scene 1), adolescents’ peers (Scene 2), and family (Scene 3)

#### Comic book characters

Mothers across the focus groups reported that the comic book should include diverse characters, including girls and boys from different races and ethnicities, feature the main character to be from the community, and show families. Mothers indicated that the main characters should look like they are “from the community.” Somali mothers had specific input on how the main character should look, saying girls should be “wearing hijab and dressed modestly.” There was a consensus across the focus groups about the importance of showing families. However, conversations diverged when discussing which family members to highlight. Somali mothers suggested grandmothers and fathers; however, Ethiopian mothers stated that the inclusion of mothers was sufficient, as shown in the mock-up. Discussing the storyline, an Ethiopian mother emphasized the central role mothers play in the family.

*Here … is Senait [the main character], Helen [character’s friend], doctor, mother, and students. Mother means and represents the whole family, the doctor is here to tell what the vaccine is … Senait is there to tell the information to her mother, what she heard from her friend...So, I don’t think it is important to include additional people in the story.*

Mothers across the focus groups also mentioned about the importance of including both boys and girls together in the story and responded positively to the exchange of information between boys and girls illustrated in the mock-up as being realistic. An Ethiopian mother said:

*[pointing to the group picture], this is effective in showing that boys share information to girls and the girls share information to boys, and prevent themselves from the disease.*

#### Information needs

Mothers expressed that they had a lack of information about HPV infection transmission, treatment, and prevention. Some mothers mentioned the importance of understanding the “risks and benefits” of the HPV vaccine in making vaccine decisions and stated that the mock-up captured these well. A Somali mother shared this sentiment by commenting on the storyline:

*I think the message here is that when you explain something to someone, they’ll understand it. The person [referring to the main character] was unsure before and thought it was a bad thing, and then … when the benefits of the shot and the dangers of the disease were explained … understood and was able to act on it and get the vaccine.*

#### Message appeal

The mothers responded positively to the deep connection between “the girl and her mother” and their affective interaction in the storyline. Ethiopian mothers also identified with the humorous twist about the pain message, with mothers bursting out laughing when the focus group moderator read that section of the script (where the main character’s friend tells the main character that getting vaccinated is an easier pain compared to studying for their next math test). Mothers emphasized that “most of the time children talk about the pain” for vaccination and responded positively that the comic book addressed pain. An Ethiopian mother described how she anticipates her child’s response to the message.

*I am very happy and excited to see this [the story]. For me the story is very good, particularly talking about the pain. My child understands a message better when communicated this way than [when] I tell him.*

#### Diffusion of information

Across all focus groups, mothers agreed that “schools play an important role” in teaching children about the HPV vaccine. Some mothers commented positively on the way the story represented HPV information diffusing from school to home - how the main character learned about the vaccine in school and used this information to engage her mother and grandmother. An Ethiopian mother said, “*Like in the story our kids communicate what they hear from school*.” An Eritrean mother shared similar sentiments about the utility of schools to promote health information since school is where children spend most of their day.

#### Social influence

Across all focus groups, mothers discussed the influence that peers have on their children. Mothers stated that their children “listen,” “feel comfortable,” and “learn better” from their peers rather than from parents. They also mentioned that their children had more access to information as they are more assimilated to western culture. An Eritrean mother explained that these differences in acculturation can create parent-child communication challenges and discussed the importance of having peers educate one another.

*Our children think we don’t know and don’t understand, and that is why they don’t listen to our advice. They don’t understand us. I really think that having young people close to their age talking to them would work best.*

Mothers agreed that if their children get information from multiple sources, it helps validate what they have heard and enhances message persuasion. Across all focus groups, mothers thought children were exposed to information from school, peers, healthcare providers, and community centers; they recommended that this was portrayed in the comic book.

### Production of the comic book

The suggestions from the focus groups were operationalized during production and included mothers’ preferences for characters, information needs for HPV and HPV vaccine, and socio-cultural beliefs (Table 3). To address information needs, we embedded the theory-based messages (messages around perceived susceptibility, severity, benefits, and barriers) created in Table 2 into the storyline to educate and promote behavior change. Messages around subjective norms were integrated into the main character’s interaction with her social environment.

**Fig. 1:**
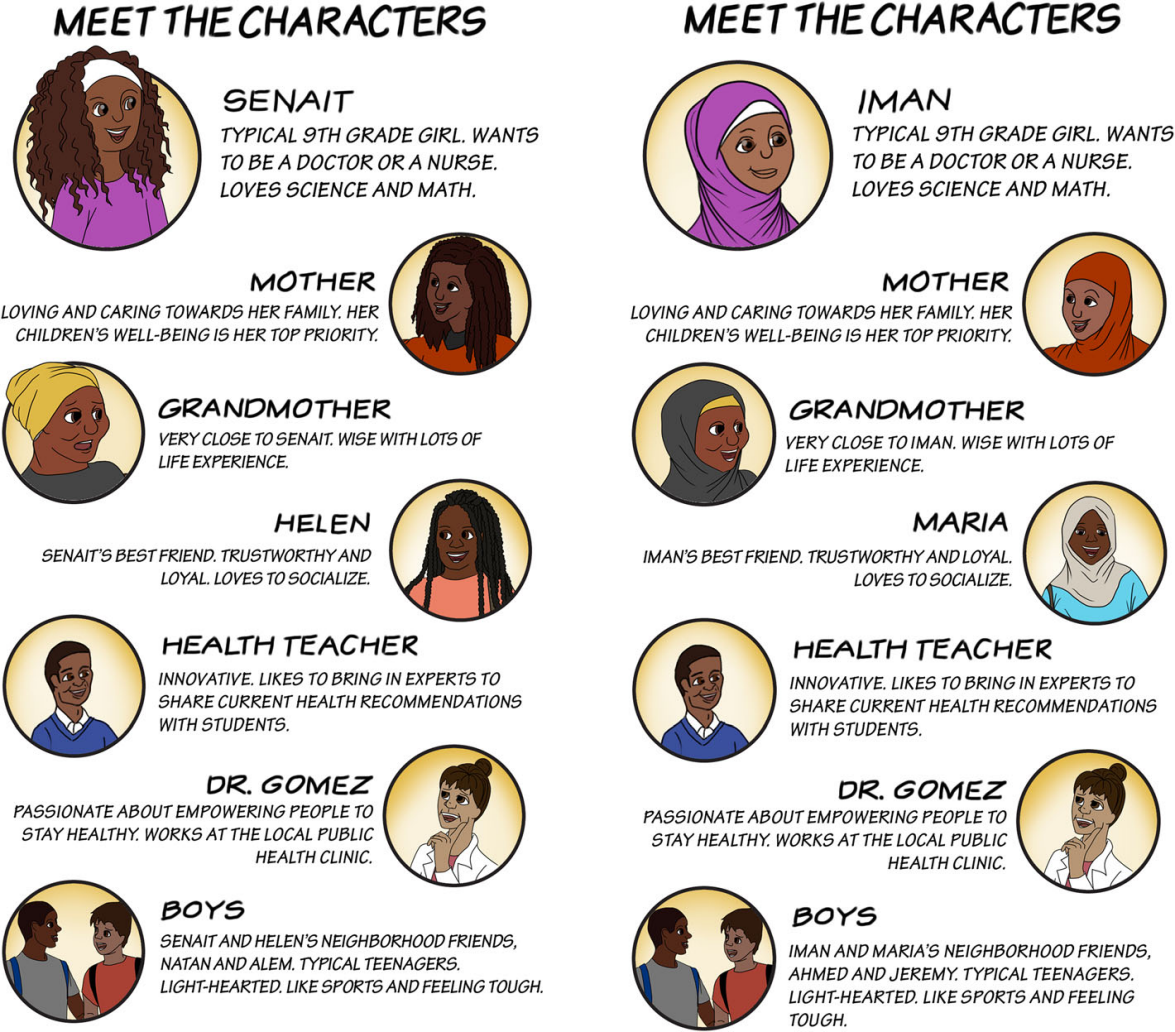
Description of the comic book characters (Image 2018; Author Isabelle Celentano). a. characters’ page from the Ethiopian comic book. b. characters’ page from the Somali comic book

**Fig. 2:**
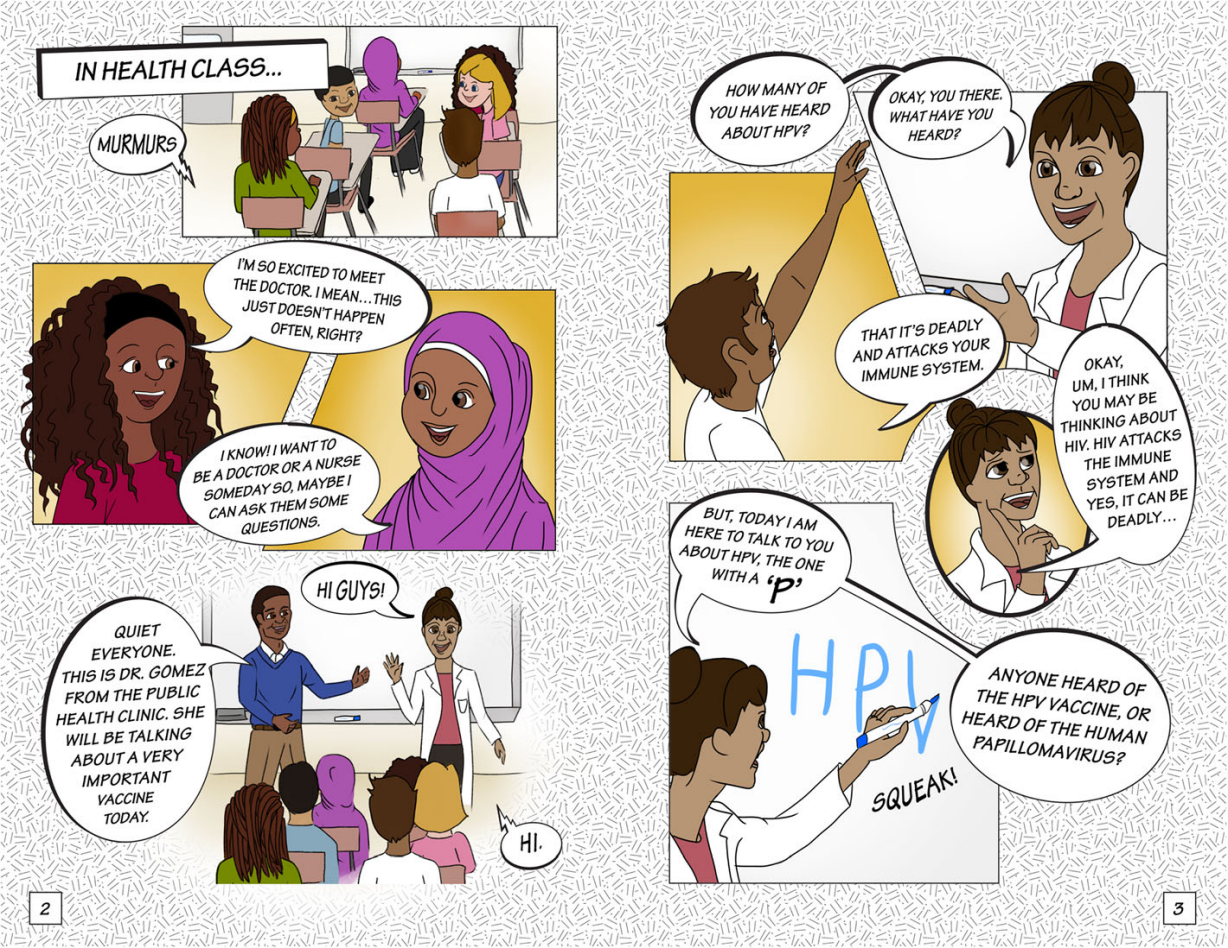
Illustrations depicting the storyline for scene 1 in the Somali comic book (Image 2018; Author Isabelle Celentano)

**Fig. 3:**
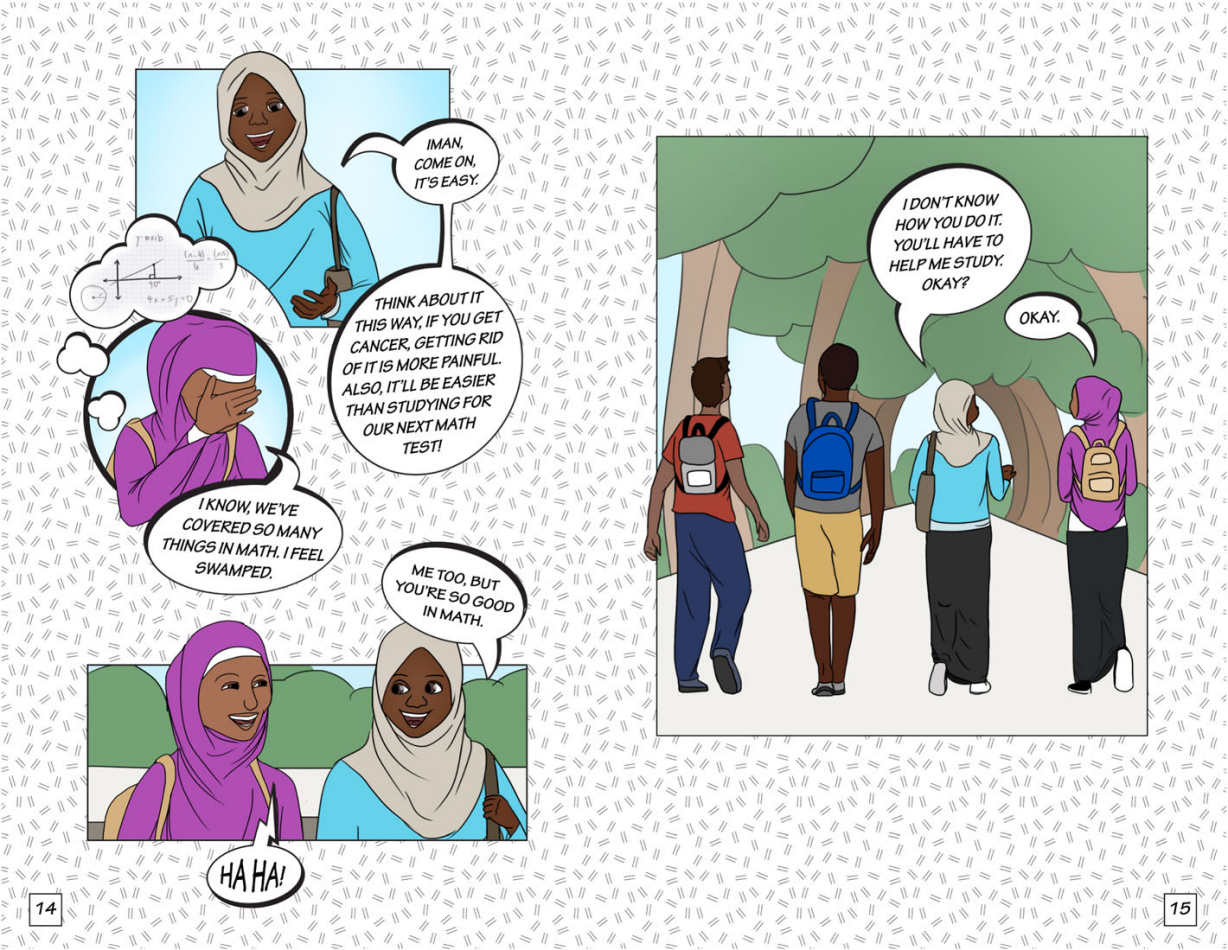
Illustrations depicting the storyline for scene 2 in the Somali comic book (Image 2018; Author Isabelle Celentano)

**Fig. 4:**
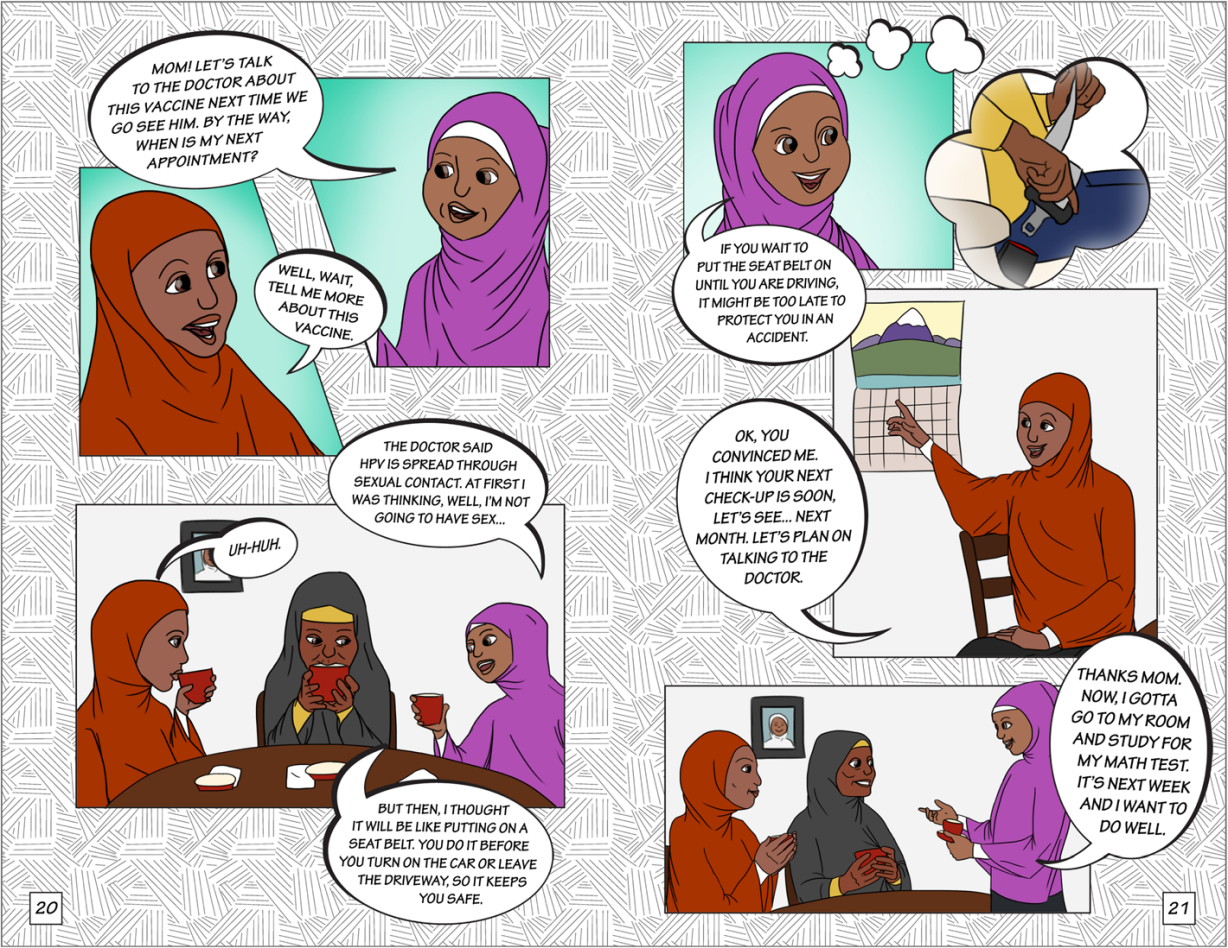
Illustrations depicting the storyline for scene 3 in the Somali comic book (Image 2018; Author Isabelle Celentano)

The final story had three scenes (Table 1). After finalizing the script for each scene, we developed the storyboard using graphic frames and dialogues, laying out conversation breaks and flow for each image. First author, IC, then sketched a draft of the main characters, beginning with the Somali version of the comic book. Following discussions with the research team and community partners, IC developed several styles and versions of the characters, which were then reviewed by the team. Figure 1 shows the final “Meet the Characters” page for the Ethiopian (Fig. 1a) and Somali (Fig. 1b) versions of the comic book. The Eritrean version had the same images as the Ethiopian comic book, but the names of the characters were different and reflective of the community. All three versions of the comic book (Ethiopian, Eritrean, and Somali) had identical dialogue. The artist created character sketches in Adobe Photoshop and finalized the comic book layout and features in Adobe Illustrator. Figures 2, 3 and 4 show illustrations from each of the three scenes of the Somali version of the comic book.

### Demographics of the adolescents

A total of 136 adolescents participated in the dinners; 134 completed an acceptability question. The mean (standard deviation) age of the 134 adolescents was 15.1 (1.1) years. Most were Somali (90.4%), few were Ethiopian (9.6%), and just over half were girls (53.7%).

### Acceptability of the comic book

Five major categories were identified including overall appeal of the comic book, structure, characters, story and content, and important messages. Most adolescents provided comments related to overall appeal (82.8%), story and content (71.6%), and important messages (73.1%), whereas fewer commented on the structure (42.5%) or characters (38.0%) (Table 4). Overall, the comic book appealed positively to most adolescents who commented on this category (82.9%), with a few reporting neutral (7.2%) or negative (9.9%) responses. Comments about structure included positive responses to the graphics (29.8%) and ease of comprehension (38.6%). Adolescents who commented on the structure were evenly split about the length; half thought the length was appropriate, while the other half thought it was too long. Comments about the characters were mostly positive; respondents noted the ethnic representation (47.1%) and diversity of the characters (13.7%). A few adolescents reported that characters did not seem realistic (3 out of 20 who commented on this code), while the remaining 17 found the characters to be relatable. Comments around the story and content were all positive, with most of these responses noting the educational value of the story (97.9%), followed by humor appeal (35.4%) and the flow of information among peers (32.3%). Finally, adolescents who commented on important messages noted that these included information on the HPV vaccine and the importance of receiving HPV vaccination (83.7%), seeking social support (e.g., how to talk with parents) (43.9%), and information about HPV and cancer (33.7%).

**Table 4:** Comic book acceptability based on the survey comments (*N* = 134^a^)

Categories	n	%^**b**^	Example comments
***Overall appeal (n = 111, 82.8%)***			
Positive	92	82.9	• I liked everything; it was very good reading.
Neutral	8	7.2	• I liked how the [comic book] explained it, but … didn’t like that the characters seem clueless.
Negative	11	9.9	• Horrible comic; I could’ve done better.
***Comic book structure (n = 57, 42.5%)***			
Images	17	29.8	• Illustration was beautiful, and I learned a lot.
Length	18	31.6	• Yes, because it’s fun to read, and it’s a short story … and it explains a lot. • I didn’t like … that they talked too much, and [it was] a lot of reading.
Comprehension	22	38.6	• It’s easy to understand what is happening in this book, and HPV is different than HIV.
***Characters (n = 51, 38.0%)***			
Ethnic Representation	24	47.1	• I liked the characters because you never see dark-skinned Muslims in comics. • I like … the Somali characters.
Diverse characters	7	13.7	• That Iman is thinking about HPV and talking to her mother and grandmother about [it].
Realistic/relatable	20	39.2	• It shows people who are similar to them and can relate to it. • I didn’t think it was realistic … the mom is easily convinced as well as the students.
***Story and Content (n = 96, 71.6%)***			
Education/information	92	95.8	• I thought it was nice, it taught me a lot about HPV [that] I didn’t know.
Information sharing	31	32.3	• I like [that] the friends talk to each other about their vaccination.
Humor	34	35.4	• I liked how it was funny.
***Important messages (n = 98, 73.1%)***			
HPV	33	33.7	• HPV can lead to cancer.
HPV vaccine	82	83.7	• Get yourself vaccinated before it’s too late.
Perceived barriers	11	11.2	• To not be scared of needles. • I liked how the book highlighted that the HPV vaccine doesn’t have pork gelatin because I know I [would] get it [without] this barrier.
Social support	43	43.9	• It taught me to talk to my parents about HPV and how I could prevent from getting it.

^a^Two of the 136 adolescents who participated in the intervention did not complete the acceptability feedback form

^b^The denominator for each percentage is the number of adolescents who commented on the main theme

## Discussion

This paper presents the process for developing and producing a theory-based HPV comic book for East African adolescents in the US. We used a multi-step process involving theories, review of the literature, and focus group data to understand socio-cultural beliefs around the HPV vaccine and participants’ input on an HPV comic story mock-up. This process enabled us to identify “what” to communicate to adolescents to promote behavior change and “how” to communicate in a way that increases understanding and appeal for both mothers and adolescents.

Our study shows that input from mothers was informative for creating comic books that depict culturally appropriate graphics, realistic content, and that integrate humor appeal. Mothers across the focus groups helped us see the need for two different sets of graphic images with characters tailored to Somali, Ethiopian, and Eritrean communities to enhance cultural relevance. Given the stigmatization of sexual health in East African communities [24, 25], the focus groups with mothers ensured that the content of the comic book was “parent approved” prior to showing it to their children. The adolescents corroborated mothers’ suggestions about ethnic representation and the rare experience of receiving a comic book with characters that resembled them. The use of characters that represent members of the community has been noted in the literature to help individuals connect with the characters [1, 33] and promote observational learning by increasing the credibility of the characters as role models [5, 34].

Mothers helped us realistically depict the relationship between peers, mothers and daughters, and the exchange of information on HPV vaccination. Mothers stated the importance of including both boys and girls in the comic book and the exchange of HPV information between them. This validates the literature that shows peers’ influence [35] on behaviors that adolescents perceive having more control over [36]. Additionally, although past studies describing the flow of information between parents and their children were reported to be “top-down” [37, 38], studies among immigrant families have shown that children often have more access to information and understanding of available resources than their parents [38, 39] and that information flow is bidirectional between parents and children, corroborating the findings from our study.

Our findings show that pain is a notable children’s concern that needs to be addressed in health education. Pain has been identified as a major barrier for children and adolescent vaccines [40]. In our comic book, we addressed pain by introducing humor; a friend of the main character compares the pain of getting the HPV vaccine to the “pain” of studying for their next math test. This approach to pain resonated with mothers, who agreed that the storyline was a realistic depiction of their children’s concerns. It also resonated with adolescents, where some noted humor as what they liked about the content. Humor is used for enhancing the resonance of health messaging, and they are effective when highlighting positive feelings and benefits of a health behavior [34]. In our study, it is possible that humor worked to both diffuse the concerns about pain and sexual stigma by promoting positive feelings of the benefits of HPV vaccine for cervical cancer prevention [34, 41].

A key strength of this study is its rigorous, multi-step approach used to create the comic book and assess its acceptability among East African adolescents. The multi-step process that we titled “message mapping” enabled us to integrate findings from focus groups, map these findings to theory, and create a comic book that addresses adolescents’ concerns and simultaneously appeal to adolescents and their parents. Additionally, to our knowledge, this is the first HPV vaccine-focused health communication that uses a comic book for East African adolescents.

There are several limitations. First, this study relied on the input of East African mothers and did not include adolescents’ input in the formative phase. This step was necessary to ensure that the content was approved by mothers before we presented it to their children. Children had the opportunity to provide input on the comic books after the comic books were developed. The second limitation is the lack of generalizability of our findings as this study is specific to the East African communities residing in the Seattle metropolitan area. Third, we were not able to evaluate the acceptability of the comic book among Eritrean adolescents and most participants were from the Somali community.

## Conclusion

Promotion of HPV vaccine uptake among East African communities needs to consider adolescents’ perspectives and concerns. Our study show that comic books are an innovative health communication medium that can educate adolescents on sensitive topics, in an entertaining way, while also appealing to parents. A rigorous multi-step process that integrates theory, empirical findings, and focus group data can help create health messages that are culturally appropriate to promote behavior change among East African communities.

## Supplementary information

**Additional file 1.** Open ended questions directed at adolescents to capture acceptability of the comic book.

## Data Availability

Data files (including raw data) and materials pertaining to this publication are available upon request to the corresponding author, Linda K. Ko, at lindako@uw.edu

## Abbreviations

HPV: Human Papillomavirus
HBM: Health Belief Model
