# Supplementary material for: Development of a theory-based HPV vaccine promotion comic book for East African adolescents in the US
Source: BMC Public Health. 2021 Jun 14;21:1137. doi: 10.1186/s12889-021-11005-2 (PMC8201717; doi:10.1186/s12889-021-11005-2)
Supplement: Supplementary file 1 — Additional file 1. Open ended questions directed at adolescents to capture acceptability of the comic book. [file 12889_2021_11005_MOESM1_ESM.docx]

Additional file 1. Open ended questions directed at adolescents to capture acceptability of the comic book

We would like to find out what you thought about the comic book. Please read each question below and write your response in the space provided. There are no right or wrong answers. We are interested in your opinions.

| **What is your general impression of the comic book?** |
| --- |
| **What did you like about it? What did you not like about it?** |
| **Do you think this comic would appeal to other adolescents? Why or why not?** |
| **What are some messages in the comic that were important to you?** |
